# Succession of Microbial Communities and Identification of Indigenous PAH‐Tolerant Bacteria Under Multiple PAH Stresses in Mangrove Sediments From Maowei Sea, Guangxi, China

**DOI:** 10.1111/1751-7915.70467

**Published:** 2026-11-10

**Authors:** Yunru Li, Songze Chen, Houxian Lan, Yun Tao, Qing Li, Xian Xu, Ju Huang, Ting Su, Xiyu Zhang, Tianqi Li, Dan Sun, Mingzhong Liang, Jingjing Song, Bin Gong, Ying Liu, Rongping Bu

**Affiliations:** ^1^ Pinglu Canal and Beibu Gulf Coastal Ecosystem Observation and Research Station of Guangxi, Guangxi Key Laboratory of Marine Environmental Disaster Processes and Ecological Protection Technology, College of Marine Sciences Beibu Gulf University Qinzhou China; ^2^ Shenzhen Ecological and Environmental Monitoring Center of Guangdong Province Shenzhen China; ^3^ Guangxi Institute of Oceanography Ecological Environment Monitoring Service co., Ltd. Beihai China

**Keywords:** bioremediation, mangrove sediment, microbial community shifts, PAH‐ tolerant bacteria, polycyclic aromatic hydrocarbons

## Abstract

Elucidating microbial community shifts under polycyclic aromatic hydrocarbon (PAH) stress and screening indigenous PAH‐tolerant microbial resources are valuable for exploring potential bioremediation strategies for PAH‐contaminated mangrove ecosystems. In this study, surface sediments were collected from mangrove wetlands of the Maowei Sea, Guangxi, China. Three rounds of successive 7‐day enrichment cultures were performed with gradually elevated PAH concentrations using phenanthrene, anthracene, and pyrene as target carbon substrates, followed by isolation and taxonomic identification of PAH‐adapted bacteria. The results demonstrated that microbial community diversity decreased with increasing enrichment rounds and varied significantly among different PAH substrate treatments. ANOSIM confirmed significant differences in community structure across enrichment cycles and PAH treatments (*p* < 0.01), consistent with the ordination patterns revealed by Non‐metric Multidimensional Scaling (NMDS). The PAH‐adapted microbial communities were predominantly dominated by Pseudomonadota (33.2%–99.9%), Bacillota (0%–48.3%), and Actinomycetota (0.1%–33.7%). In total, 17 indigenous PAH‐tolerant bacterial strains belonging to eight genera within the phylum Pseudomonadota were isolated, with representative strains affiliated with *Labrys*, *Sphingomonas*, and *Acinetobacter*. Within the tested physiological gradient ranges, *Labrys* sp. F3‐1 and *Acinetobacter* sp. B1‐5 exhibited optimal growth at pH 5.0, 40°C, and 15‰ salinity, while *Sphingomonas* sp. N2‐15 showed the best growth performance at pH 5.0, 25°C, and 4‰ salinity. Growth assays under PAH pressure further verified that these three isolates could survive and proliferate in PAH‐amended selective media under the tested laboratory conditions. This study provides indigenous PAH‐tolerant bacterial resources and fundamental community‐level insights for future investigations on microbial‐based remediation of PAH‐polluted mangrove sediments. Notably, the high PAH concentrations applied during laboratory enrichment (100–500 mg/L) were substantially higher than in situ environmental levels; the observed microbial responses and strain physiological traits reflect adaptive characteristics under extreme laboratory selective pressure and should be cautiously extrapolated to natural mangrove ecosystems.

## Graphical abstract

Significantly discrepancies in microbial communities across different PAH substrates and enrichment cycles (*p* < 0.01) were observed, with Pseudomonadota (33.2%–99.9%), Bacillota (0%–48.3%), and Actinomycetota (0.1%–33.7%) dominating PAH‐tolerant bacteria. Notably, *Labrys* sp. F3‐1, *Acinetobacter* sp. B1‐5, and *Sphingomonas* sp. N2‐15 represent novel acidophilic, mesophilic, and (moderately) halophilic strains that can tolerate phenanthrene, anthracene, and pyrene, respectively, as their sole carbon and energy sources.

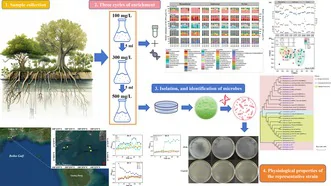

## 1 Introduction

Polycyclic aromatic hydrocarbons (PAHs) are typical refractory organic pollutants composed of multiple condensed aromatic rings. They predominantly stem from imperfect fossil fuel combustion plus diverse anthropogenic pollution (Lindström et al. 2021; Souza et al. 2022; Yilmaz et al. 2022; Li et al. 2023). Owing to their high hydrophobicity, environmental persistence and strong bioaccumulation capacity, PAHs can induce mutagenesis and carcinogenesis, posing serious threats to environmental equilibrium and public wellness (Nowakowski et al. 2021; Barbosa Jr et al. 2023; Altarawneh and Ali 2024). As essential coastal blue‐carbon ecosystems, mangrove wetlands feature high primary productivity and powerful sediment retention capability, and thus serve as crucial natural repositories of diverse persistent organic pollutants (POPs) (Lin et al. 2019; Alhassan and Aljahdali 2021; Dobslaw et al. 2021; Girones et al. 2021; Yu et al. 2023; Lu et al. 2025).

Widespread PAH contamination has been documented in mangrove wetlands worldwide, such as Maowei Sea (with a maximum PAH content of 138.1 × 10^−9^ ng/g dry weight (dw)) (Yan et al. 2022), Zhenzhu Bay (28.06 ng/g dw) (Pang et al. 2023), Beihai (320.07 ng/g dw) (Gao et al. 2026), Zhanjiang mangrove (404.8 ng/g dw) (Yan et al. 2023), Shenzhen (5168 ng/g dw) (Li et al. 2014), Zhangjiang Estuary (247.76 ng/g dw) (Zhang, Li, et al. 2025; Zhang, Qian, et al. 2025), Yueqing Bay (270.6 ± 21.1 ng/g dw) (Huang et al. 2022), Hong Kong MaWan mangrove (400.7 ng/g dw) (Wang et al. 2015), Dongzhai Harbour (1481.91 ng/g dw) (Zheng et al. 2025), Bodo in Nigeria (2350 ng/g dw) (Saunders et al. 2022), and Sundarbans Mangrove (2 × 10^4^ ng/g dw) (Balu et al. 2020). Although this background PAH level is relatively low compared with heavily polluted sites such as Beihai, long‐term low‐level PAH input from shipping, aquaculture and land‐based runoff has imposed sustained mild selective pressure on indigenous microbes (Han et al. 2022). Native strains acclimated to local sediments are more suitable for prospective in situ bioremediation than exotic isolates from highly polluted areas. Moreover, Maowei Sea mangrove is a key protected wetland in Guangxi facing potential PAH accumulation risks, and this site is representative of subtropical mangroves in the northern Beibu Gulf (Wang et al. 2017).

Microbial bioremediation, which leverages natural microbially driven degradation of recalcitrant organic contaminants, is an economical, environmentally benign and long‐term viable remediation technology that imposes slight disturbance on the core ecological functions of soil and marine environments (Davletgildeeva and Kuznetsov 2024; Li et al. 2025; Ma et al. 2025). So far, indigenous PAH‐degrading bacteria have been widely recovered from mangrove ecosystems, terrestrial soils, estuaries and marine environments (Zhou et al. 2020; Zhang, Manzoor, et al. 2021; Zhang, Qiu, et al. 2021; Alotaibi et al. 2024; Brzeszcz et al. 2024; Li, Cao, et al. 2024; Li, Huang, et al. 2024; Lu et al. 2026). These isolates mainly belong to three dominant bacterial phyla: Pseudomonadota (e.g., *Aquabacter*, *Roseovarius*), Actinomycetota (*Mycolicibacterium*), and Bacillota (*Bacillus*) (Zhou et al. 2020; Brzeszcz et al. 2024; Lu et al. 2026). As a typical case, *Aquabacter sediminis* P‐9ᵀ can completely degrade 50 mg/L phenanthrene (PHE) in under 5 days. *Roseovarius* sp. SCSIO 43702 achieves a 96% removal efficiency of PHE with an initial loading of 100 mg/L over 10 days. In addition, *Bacillus halotolerans* exhibits a maximum degradation efficiency of over 50% against benzo[a]pyrene (BaP). The core genes closely linked to verified PAH biotransformation include *nid*, *phd*, *nah* and *pah* (Sakshi and Haritash 2020, Hua et al. 2023), and the corresponding metabolic pathways are the salicylate, phthalate and gentisate pathways (Zhang, Manzoor, et al. 2021; Zhang, Qiu, et al. 2021; Li, Cao, et al. 2024; Li, Huang, et al. 2024; Lu et al. 2026).

At present, Maowei Sea mangrove wetlands have suffered widespread PAH contamination (Yan et al. 2022), owing to diverse human‐related activities including shipping; however, research on the bioremediation of these PAH‐polluted mangroves remains limited. To address this research gap, we harvested top‐layer sediments from the Maowei Sea Mangrove Reserve in Guangxi and performed three rounds of 7‐day gradient enrichment cultivation (substrate concentrations of 100, 300, and 500 mg/L, respectively), in which phenanthrene, anthracene, and pyrene were separately used as the sole carbon sources. The present research therefore seeks to investigate (1) the shifts in diversity and community composition of microbial consortia across different enrichment cycles and PAH substrates; (2) to isolate and taxonomically identify indigenous bacterial strains capable of growing in PAH‐amended media after the third round of enrichment; and (3) to screen three representative strains with differential growth performance on PHE, ANT, and PYR, followed by profiling their physiological growth traits along with growth tests under PAH‐stressed culture conditions. The findings provide valuable microbial resources and theoretical support for the microbial remediation of PAH‐contaminated mangrove sediments.

## 2 Materials and Methods

### 2.1 Site Description and Sediment Collection

Surface sediments at a depth of 0–5 cm were sampled from five sites (MWH1–MWH5) within the Maowei Sea mangrove wetland in Guangxi, China, on 10 October 2024. Geographic positions of the study area and sampling locations are illustrated in Figure 1, with full details given in Table S1. The five sampling sites were selected based on anthropogenic disturbance gradients and spatial distribution, with uniform elevation and tidal conditions to eliminate topographic interference. Each site adopted composite sampling by mixing multiple subsamples within a 5 m × 5 m quadrat. Independent field triplicates were not collected due to practical constraints, and this limitation is declared explicitly in the manuscript.

**FIGURE 1:**
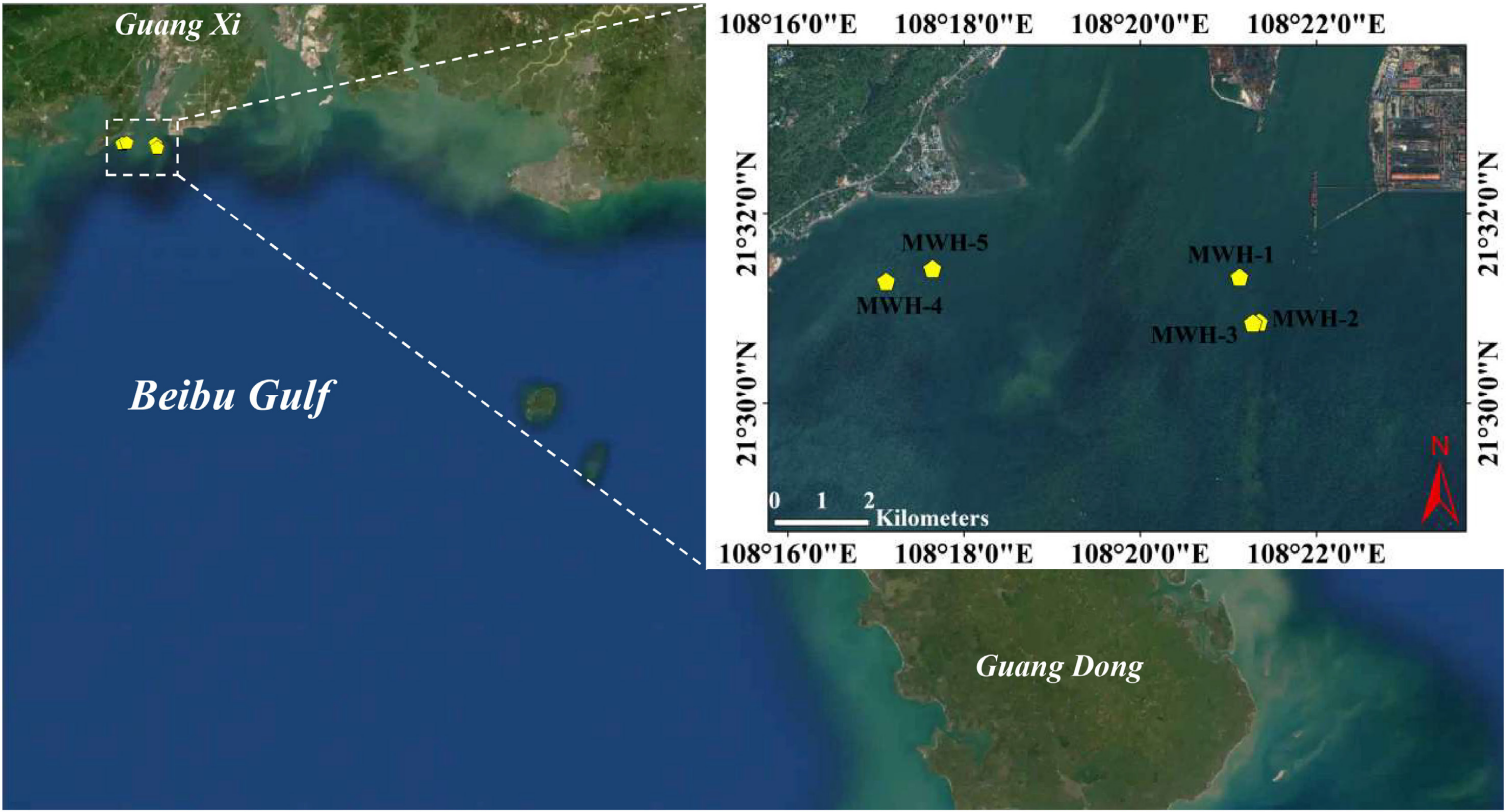
Location of study area and sampling sites (marked by yellow regular pentagon) in the Maowei Sea mangrove wetland, Guangxi, China. Sampling sites: MWH‐1, MWH‐2, MWH‐3, MWH‐4, and MWH‐5.

All samples were collected aseptically into sterile sampling bags, stored on ice and shipped to the laboratory in less than 24 h for further analysis. During sampling, the in situ temperature, pH, salinity, and moisture content of sediments were measured on site by a portable integrated physicochemical analyser.

### 2.2 Successive Enrichment Culture of PAH‐Tolerant Microorganisms

Approximately 0.2 g each of phenanthrene (PHE, ≥ 99%), anthracene (ANT, ≥ 98%), and pyrene (PYR, ≥ 97%) were weighed and dissolved in 100 mL of acetone (≥ 99.5%) to prepare stock solutions with a final concentration of 2 g/L for each compound. All reagents were purchased from Macklin Biochemical Co. Ltd., China. Subsequently, these solutions underwent filtration via sterile hydrophobic polytetrafluoroethylene (PTFE) membrane filters (0.2 μm pore size) and a diameter of 25 mm (Millipore Express) to remove microbial contaminants. Obtained filtrates were preserved inside sterile brown glass vials at 4°C prior to subsequent experimentation.

Aliquots of 20 g fresh surface sediment (0–5 cm depth) were introduced into 180 mL of sterile mineral salt medium (MSM) (Zhang et al. 2019; Kashif et al. 2023). The basal MSM components included 0.27 g/L K_2_HPO_4_, 0.35 g/L KH_2_PO_4_, 2.7 g/L NH_4_Cl, 0.1 g/L MgCl_2_·6H_2_O, and 0.1 g/L CaCl_2_·2H_2_O, and underwent autoclaving at 121°C over a 20‐min sterilization cycle before utilization.

Subsequently, 1 mL of vitamin mixture and 1 mL of trace element solution were added to the medium with a final dilution of 1:1000 (v/v). The vitamin mixture comprised 20 mg/L D‐biotin, 20 mg/L folic acid, 100 mg/L pyridoxine hydrochloride, 50 mg/L thiamin hydrochloride, 50 mg/L nicotinic acid, 50 mg/L DL‐calcium pantothenate, 50 mg/L 4‐aminobenzoic acid, and 10 mg/L vitamin B12. The trace element solution contained 9 mg/L FeCl_2_·4H_2_O, 4 mg/L MnCl_2_·4H_2_O, 1.4 mg/L ZnCl_2_, 1 mg/L CoCl_2_·6H_2_O, and 1 mg/L (NH_4_)₆Mo₇O_24_·4H_2_O. Both solutions were disinfected using 0.2 μm PES membrane filters before addition. Then, 10 mL of 2 g/L PHE, ANT, or PYR solution was added separately to each culture system as the target carbon substrate to yield a final loading of 100 mg/L. Acetone was allowed to evaporate completely before supplementation. Medium pH was titrated to 7.4, and the salinity was kept at 4‰.

All primary enrichment cultures were incubated in the dark under 25°C and continuous orbital shaking (180 rpm) for 7 days. Enrichment was performed for three consecutive rounds, and the PAH concentration was gradually increased to 300 mg/L for the second round and 500 mg/L for the third round. At each transfer, 5 mL of culture broth from the prior round was introduced into 180 mL fresh sterile MSM amended with PAHs at designated concentrations, and each enrichment cycle lasted 7 days. All treatments were conducted with three biological replicates.

Three samples, namely N2–1, N3–1, and B2–3, were discarded during enrichment incubation owing to cracked Erlenmeyer flasks and excluded from subsequent analyses. Specifically: N2–1 denotes ANT as the sole carbon source, MWH–2 as the sampling site, and −1 as the first 1‐week enrichment cycle; N3–1 refers to ANT as the sole carbon source, MWH–3 as the sampling site, and −1 as the first 1‐week enrichment cycle; B2–3 stands for PYR as the sole carbon source, MWH–2 as the sampling site, and −3 as the third 1‐week enrichment cycle.

Notably, the gradient elevated PAH concentrations (100–500 mg/L) were designed to impose strong directional selective pressure to screen indigenous PAH‐tolerant bacteria, rather than simulating the natural background PAH concentration of Maowei Sea mangrove sediments (approximately 138.1 ng/g dry weight; Yan et al. 2022). Low ambient PAH levels cannot provide sufficient stress to suppress the massive proliferation of ubiquitous heterotrophic bacteria that utilize natural organic carbon, which would hinder effective enrichment of target PAH‐adapted strains (Ashok and Agusti 2022; Peng et al. 2026). This gradient high‐stress enrichment strategy is widely adopted in previous studies for isolating PAH‐resistant bacteria from coastal wetland sediments (Du et al. 2025; Lu et al. 2026). Furthermore, the fixed PAH concentration of 500 mg/L was later applied in the physiological gradient tests (pH, temperature, salinity) of representative isolates to guarantee unified stress intensity for horizontal comparison of strain tolerance traits. The physiological characteristics determined under such high PAH stress only reflect the anti‐stress performance under laboratory extreme conditions, and the adaptability of these strains under environmentally relevant low PAH concentrations requires further verification in subsequent research (Vogel et al. 2023). It should be noted that vitamins and trace elements were supplemented in the MSM medium. Although these additives were supplied at very low concentrations, they may contain trace organic carbon. In addition, serial transfers during enrichment could introduce minor carry‐over organic substances from previous cultures. Therefore, PHE, ANT, and PYR are referred to as target carbon substrates in this study, rather than the sole carbon source. The enrichment system was used to select strains tolerant to PAH stress, and further controlled experiments will be performed to confirm PAH utilization.

### 2.3 Isolation, Screening and Taxonomic Identification of Newly Isolated PAH‐Tolerant Bacteria

Solid MSM medium was prepared by adding 1.5% (w/v) agar (Solarbio, China) to the liquid MSM described above. The medium was subjected to 20 min autoclaving at 121°C and cooled before use. Each solid MSM agar plate was pre‐coated with 1 mL of 500 mg/L PHE, ANT, or PYR, respectively (hereinafter referred to as MSM‐PAH‐agar plates), with these compounds added as the target carbon substrate for bacterial enrichment and screening. Acetone was allowed to evaporate completely before use. We note that intermediate metabolites from PAH degradation in the prior enrichment may be carried over during serial transfer, and their interference to the next round of enrichment cannot be excluded at present. Thus, PHE, ANT and PYR were supplied as target carbon substrates for selective screening, and this experimental setup was not designed to verify that PAHs were the sole available carbon source. Such confounding factors will be refined in future work.

After three rounds of enrichment, 10 μL of turbid culture from each PAH treatment were serially diluted with sterile ddH_2_O to yield 10^−3^ and 10^−4^ dilutions. 100 μL of each dilution was dispensed onto MSM‐PAH‐agar plates and cultivated under dark conditions at 25°C over a 7‐day incubation period. Individual colonies were picked and purified by repeated streak isolation to obtain pure cultures. For preservation, 750 μL of each bacterial suspension was mixed with glycerol to reach a terminal concentration of 50% (v/v), and the mixtures were stored at −80°C.

For strain identification, single colonies were resuspended in 10 μL sterile ddH_2_O and heat‐lysed at 95°C for 10 min using a thermal cycler (BIOER, China). Nearly complete bacterial 16S rRNA gene fragments were amplified using the universal primer set consisting of 27F (5′‐AGAGTTTGATCMTGGCTCAG‐3′) and 1492R (5′‐TACGGYTACCTTGTTACGACTT‐3′) (Sano et al. 2021). The 50 μL PCR reaction system consisted of 25 μL of 2 × Taq PCR Master Mix (Sangon, Shanghai), 1 μL of each primer (10 μM, Sangon, Shanghai), 1.0 μL of bacterial lysate template, and 22 μL of nuclease‐free water.

The PCR cycling parameters were configured as detailed below: initial denaturation at 95°C for 3 min; 35 cycles of 95°C for 15 s, 55°C for 30 s, and 72°C for 90 s; and a final extension at 72°C for 10 min. The amplicons were visualized using 1% (w/v) agarose gel electrophoresis at 120 V for 20 min, and then sent to Sangon Biotech (Shanghai) Co. Ltd. for Sanger sequencing. The 16S rRNA gene sequences of all strains were searched against the NCBI nucleotide database via BLASTN to resolve phylogenetic relationships among these newly isolated isolates.

### 2.4 Growth Characteristics and PAH Utilization Potential of Three Representative Bacterial Strains

Three representative strains, namely *Labrys* F3–1, *Sphingomonas* N2‐15 and *Acinetobacter* B1‐5, were selected to characterize their growth traits and PAH utilization potential. We measured their optimal growth temperature at 4°C, 25°C and 40°C, while pH and salinity were kept at 7.4‰ and 4‰, respectively. Subsequently, the optimal salinity was screened at gradients of 4‰, 8‰, 15‰ and 30‰ while maintaining 25°C and a stable pH value of 7.4. The strains were cultivated at pH 5, 7, 9 and 11 to explore their growth pH range, with temperature set to 25°C and salinity to 4‰. Each assay was set up in biological triplicates and cultured at 180 rpm under dark shaking conditions for 21 days. Cell proliferation was tracked via OD_600_ quantification with a FlexA‐200 microplate spectrophotometer (Aosheng, China).

To evaluate the PAH tolerance and growth adaptability of the isolates, three representative strains were inoculated onto MSM‐PAH agar plates (treatment), PAH‐free MSM agar plates, and acetone solvent control plates, with all assays performed in triplicate (Figures 6, 7, 8 and S2). PAH stock solutions were prepared in acetone; equal acetone volumes were added to solvent controls and fully evaporated prior to pouring plates to exclude solvent interference. This study did not conduct quantitative PAH degradation tests. These PAH‐containing plates served only for qualitative screening of strain growth under PAH selective pressure, not for quantifying PAH removal or degradation efficiency. Uninoculated PAH‐containing controls were not included in this qualitative plate assay because quantitative measurement of PAH removal was not performed owing to experimental limitations. A full set of controls, including uninoculated PAH‐containing groups, will be adopted in our ongoing quantitative degradation experiments to distinguish biological transformation from abiotic PAH loss.

### 2.5 Genomic DNA Isolation, PCR Assay and Illumina‐Based High‐Throughput Sequencing

After three successive enrichment cultures, 50 mL aliquots were withdrawn from each treatment and pelleted via centrifugation at 14,000 × *g* for 10 min to harvest microbial biomass. Genomic DNA extraction was performed utilizing the FastDNA Spin Kit for Soil (MP Biomedical, USA) as guided by the official kit instructions. DNA concentration was determined with a Qubit 4 Fluorometer (Thermo Fisher Scientific, USA).

Amplification targeting the V4 hypervariable segment of bacterial 16S rRNA was carried out employing universal primer pair 515F (5′‐GTGYCAGCMGCCGCGGTAA‐3′) and 806R (5′‐GGACTACNVGGGTWTCTAAT‐3′) (Debnath et al. 2025). Each 50 μL PCR reaction was assembled as follows: 25 μL PCR enzyme mix, 2.5 μL of each primer (10 μM), and 20 μL DNA template. PCR reactions were processed using a GeneExplorer PCR system (BIOER, China) under the temperature settings listed below: predenaturation at 98°C lasting 30 s, followed by 35 repeating cycles (98°C for 10 s, 61°C for 20 s, 72°C for 30 s), then a terminal elongation step incubated at 72°C over 2 min. No‐template blanks together with positive reference samples were incorporated into every PCR batch.

PCR products were separated on 1% agarose gels, then recovered via the E.Z.N.A. Cycle Pure Kit (Omega, USA). After quantification using a Qubit 4 Fluorometer, all libraries were pooled at equal molar concentrations prior to paired‐end sequencing via the Illumina HiSeq 2500 PE250 platform.

### 2.6 Bioinformatic Data Processing

Raw amplicon reads were preprocessed with Cutadapt: low‐quality sequences (average Q‐score < 20) and primer sequences were eliminated (Martin 2011). The resulting clean sequences were subjected to truncation, denoising and chimera removal using DADA2. Taxonomic classification of 16S rRNA ASVs was performed in QIIME2 (Bolyen et al. 2018) by the classify‐sklearn plugin based on the SILVA 138.2 database. Mitochondrial and unclassified ASVs were eliminated in the final filtering step.

A total of 34,429–143,287 valid reads were obtained for 42 samples. To standardize sequencing depth, all samples were rarefied to 34,429 reads. Three common alpha diversity indices (Shannon, Chao1 and ACE) were computed using the default parameters of QIIME2 (Bolyen et al. 2019).

### 2.7 Statistical Analysis

Geospatial mapping of all mangrove sampling locations was accomplished with ArcGIS 10.8.1. Venn diagram analysis was conducted via an online platform (https://www.omicstudio.cn/tool/6) to quantify the proportions of unique and shared ASVs across three enrichment rounds and three distinct PAH substrates. A neighbour‐joining (NJ) phylogenetic tree was built with MEGA v11.0 (Kumar et al. 2016), with bootstrap support calculated from 1000 replicate resamplings. Non‐metric multidimensional scaling (NMDS) was used to visualize community dissimilarity, and Analysis of Similarity (ANOSIM) was applied to test for compositional differences among PAH substrate treatments and enrichment rounds. Both NMDS and ANOSIM were calculated based on Bray–Curtis dissimilarity matrices and implemented via the PRIMER v7 package (Clarke and Gorley 2015). ANOSIM generated R statistics and *p* values for overall and pairwise group comparisons, which are summarized in the inset table of Figure 2b. Origin was employed to generate alpha diversity plots (Shannon, ACE, and Chao1), community diversity graphs, and growth curves of representative strains. A small number of enrichment cultures were discarded due to cracked flasks during incubation, resulting in an unbalanced sample set. Damaged samples were excluded directly without missing‐value imputation. ANOSIM was performed on the remaining valid dataset, and this sample loss did not alter the core conclusions of community comparison.

**FIGURE 2:**
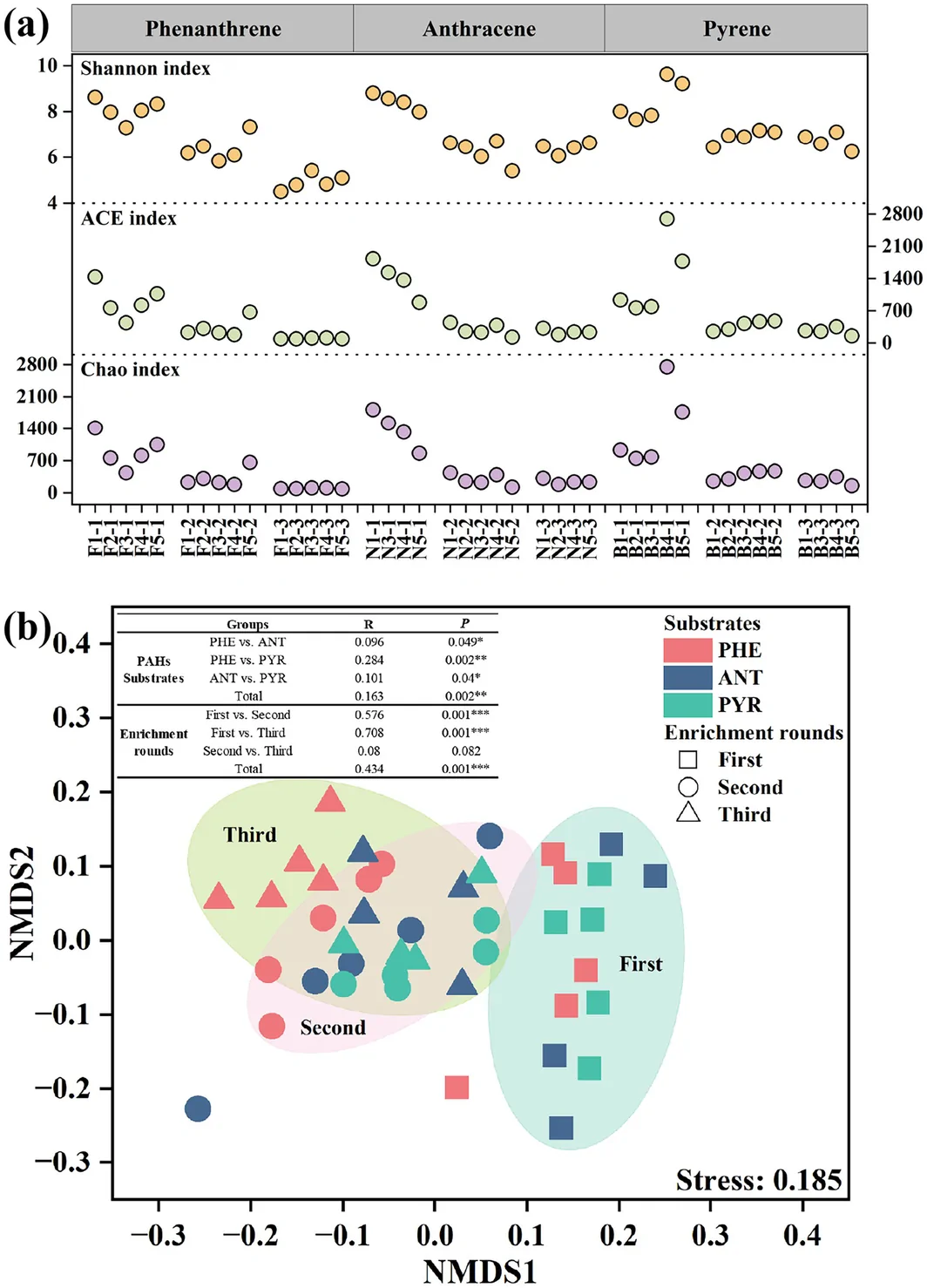
(a) Alpha diversity indices (Shannon, ACE, and Chao) of microbial communities from surface sediments of the Maowei Sea mangrove wetland after three cycles enrichment with polycyclic aromatic hydrocarbon (PAH) substrates (phenanthrene (PHE), anthracene (ANT), and pyrene (PYR)). (b) Non‐metric multidimensional scaling (NMDS) and analysis of similarities (ANOSIM) of microbial communities based on Bray‐Curtis distance metrics, grouped by the three PAH substrates (PHE, ANT, and PYR).

## 3 Results

### 3.1 Sediment Physicochemical Characteristics

Surface sediment samples were retrieved at five sampling locations across the Maowei Sea mangrove wetland, Guangxi, China. Measured sediment physicochemical indices (temperature, pH, salinity, and moisture) varied between 23.8°C and 24.6°C (mean ± SD: 24.3°C ± 0.3°C), 7.2 to 7.8 (7.4 ± 0.2), 2.3 to 4.0 psu (3.3 ± 0.7 psu), and 71.4% to 95.0% (87.5% ± 9.7%), respectively (Table S1). Basic physicochemical indicators, including in situ temperature, pH, salinity, and moisture content of sediments, were determined with standard portable instruments. The background PAH level of Maowei Sea mangrove sediments refers to the published monitoring data from previous research.

### 3.2 Alpha and Beta Diversities of Microbial Assemblages Following Three‐Round PAH Enrichment

A total of 42 surface sediment samples were obtained after three rounds of enrichment incubation with three typical polycyclic aromatic hydrocarbons (PAHs), namely phenanthrene (PHE), anthracene (ANT), and pyrene (PYR).

After quality filtration and the elimination of chimeric sequences, 2,845,262 qualified 16S rRNA reads were obtained from the entire sample set, with an average sequencing depth of 67,744 reads per sample; these valid sequences were clustered into 19,703 microbial ASVs.

Overall, microbial diversity (mainly including Shannon index) and richness (mainly consisting of ACE and Chao indices) varied with PAH substrate types (PHE, ANT, and PYR) and enrichment rounds (i.e., gradually increasing substrate concentration, 100, 300, and 500 mg/L) (Figure 2a). In addition, no obvious differences in microbial diversity and richness were observed among samples from different sampling sites. We focus on the overall changing trend of alpha‐diversity in the present work, and systematic pairwise significance comparisons between groups will be addressed in future specialized studies.

In detail, the results showed that both microbial community diversity and richness gradually decreased as enrichment rounds increased (*p* < 0.01; Figure S1). Specifically, the Shannon indices of the PHE, ANT, and PYR treatment groups decreased from 8.06 (averaged across the five sampling sites), 8.44, and 8.47 in the first enrichment cycle to 4.92, 6.40, and 6.71, respectively, by the third enrichment cycle. The PYR group exhibited higher microbial diversity than the other two groups in both the first and third enrichment cycles (Figure 2a).

On the other hand, the ACE indices for the PHE, ANT, and PYR treatment groups fell from 907.50, 1403.85, and 1393.34 at the first cycle to 96.69, 246.66, and 257.82 at the third enrichment cycle. The ANT treatment presented the highest value of the ACE index in the first cycle, whereas the PYR treatment attained the maximum richness level in the third cycle. The Chao indices displayed a comparable dynamic trend (Figure 2a).

Non‐metric multidimensional scaling (NMDS) was applied to visualize microbial community dissimilarity across PAH treatments and enrichment cycles, while analysis of similarities (ANOSIM) was used to test for compositional differences. ANOSIM revealed significant overall community divergence among PAH substrates and across enrichment rounds (*p* < 0.01), whereas pairwise comparison showed no significant difference between the second and third enrichment cycles (*p* > 0.05; Figure 2b).

### 3.3 Taxonomic Structure of PAH‐Enriched Microbiota After Three Sequential Enrichments

After the third enrichment cycle, at the phylum level, microbial communities in the PHE treatment were dominated by Pseudomonadota (76.7%–99.8%), Bacillota (0%–21.3%), and Bacteroidota (0%–1.4%). In particular, the relative abundances of Pseudomonadota and Bacillota increased from 45.0% and 0.9% in the first cycle to 94.6% and 4.3% in the third cycle, respectively, while Bacteroidota decreased from 11.8% in the first cycle to 0.3% in the third cycle (Figure 3). On the other hand, in the ANT treatment, the phylum‐level community was primarily constituted by Pseudomonadota (56.4%–77.6%), Actinomycetota (7.0%–33.6%), and Bacillota (0%–9.4%). Specifically, the relative abundances of Pseudomonadota, Actinomycetota, and Bacillota rose from 47.5%, 6.9%, and 1.6% in the first cycle to 70.9%, 19.3%, and 2.4% in the third cycle, respectively (Figure 3). Furthermore, the major microbial phyla in the PYR treatment consisted of Pseudomonadota (50.5%–65.1%), Actinomycetota (16.6%–29.3%), and Bacteroidota (7.4%–12.3%). Percentage contributions of Pseudomonadota and Actinomycetota ascended from 47.9% and 9.8% in the first cycle to 60.2% and 22.2% in the third cycle, respectively, whereas Bacteroidota fell from 10.5% in the first cycle to 9.4% in the third cycle (Figure 3).

**FIGURE 3:**
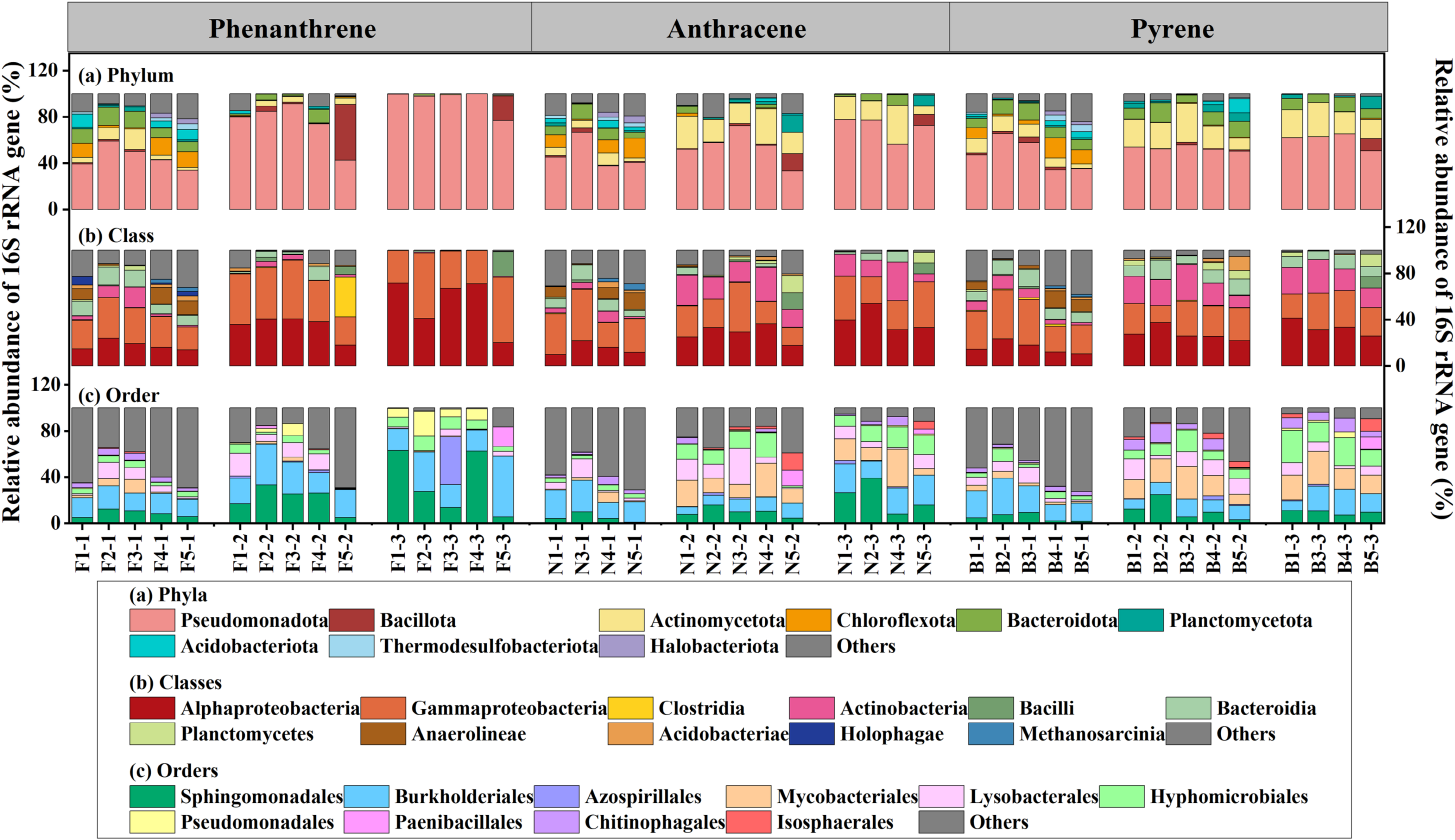
Taxonomic composition of microbial communities at three levels (phylum, class, and order) after three cycles of enrichment with PHE, ANT and PYR, respectively. Taxa with relative abundance > 5% are shown at the phylum and class levels, and the top 10 most abundant taxa are indicated at the order level. Sample designation format: [carbon source code]‐[sampling site]‐[enrichment cycle]. F = PHE, N = ANT, B = PYR; 1–5 = sampling sites 1–5; −1 to −3 = first to third one‐week enrichment cycles.

At the class level, Alphaproteobacteria (19.9%–71.8%), Gammaproteobacteria (28.0%–56.7%), and Bacilli (0%–21.3%) constituted the predominant classes in the PHE treatment. However, in the ANT and PYR treatments, the microbial community was composed of Alphaproteobacteria (31.3%–54.1% and 25.9%–41.4%), Gammaproteobacteria (22.8%–39.5% and 20.7%–31.8%), and Actinobacteria (6.8%–33.4% and 16.6%–29.0%). At the order level, Sphingomonadales (5.6%–62.9%), Sphingomonadales (8.0%–38.9%), and Mycobacteriales (15.9%–28.4%) represented the predominant microbial taxa in the PHE, ANT, and PYR treatments, respectively (Figure 3). At both family and genus levels, Sphingomonadaceae and *Sphingobium* were the predominant taxa in the PHE and ANT treatments, whereas Mycobacteriaceae and *Mycobacterium* dominated the PYR treatment (Figure S3).

Two Venn diagrams were constructed to illustrate the shared and unique ASVs among three enrichment cycles or three PAH treatments (Figure 4). The first, second, and third enrichment cycles consisted of 14, 15, and 13 samples, corresponding to 14,121, 4219, and 1980 ASVs, respectively. Venn analysis identified a total of 117 shared ASVs (0.6%), while the unique ASVs of the three cycles accounted for 13,792 (70.0%), 3777 (19.2%), and 1634 (8.3%), respectively (Figure 4a). On the other hand, the sample numbers and corresponding ASVs for PHE, ANT, and PYR treatments were 15 and 5735, 13 and 6842, and 14 and 8050, respectively. Venn analysis revealed 94 shared ASVs (1.0%) were detected among the three groups, and the unique ASVs in the three treatments accounted for 5250 (26.6%), 6292 (31.9%), and 7431 (37.7%), respectively (Figure 4b). Consistent with this marked reduction in ASV numbers across successive enrichment cycles, the proportion of “Others” at the family and genus levels gradually decreased. This trend indicates that continuous PAH selection filtered out a large number of low‐abundance, rare microbial taxa, and the community gradually converged toward PAH‐adapted dominant populations.

**FIGURE 4:**
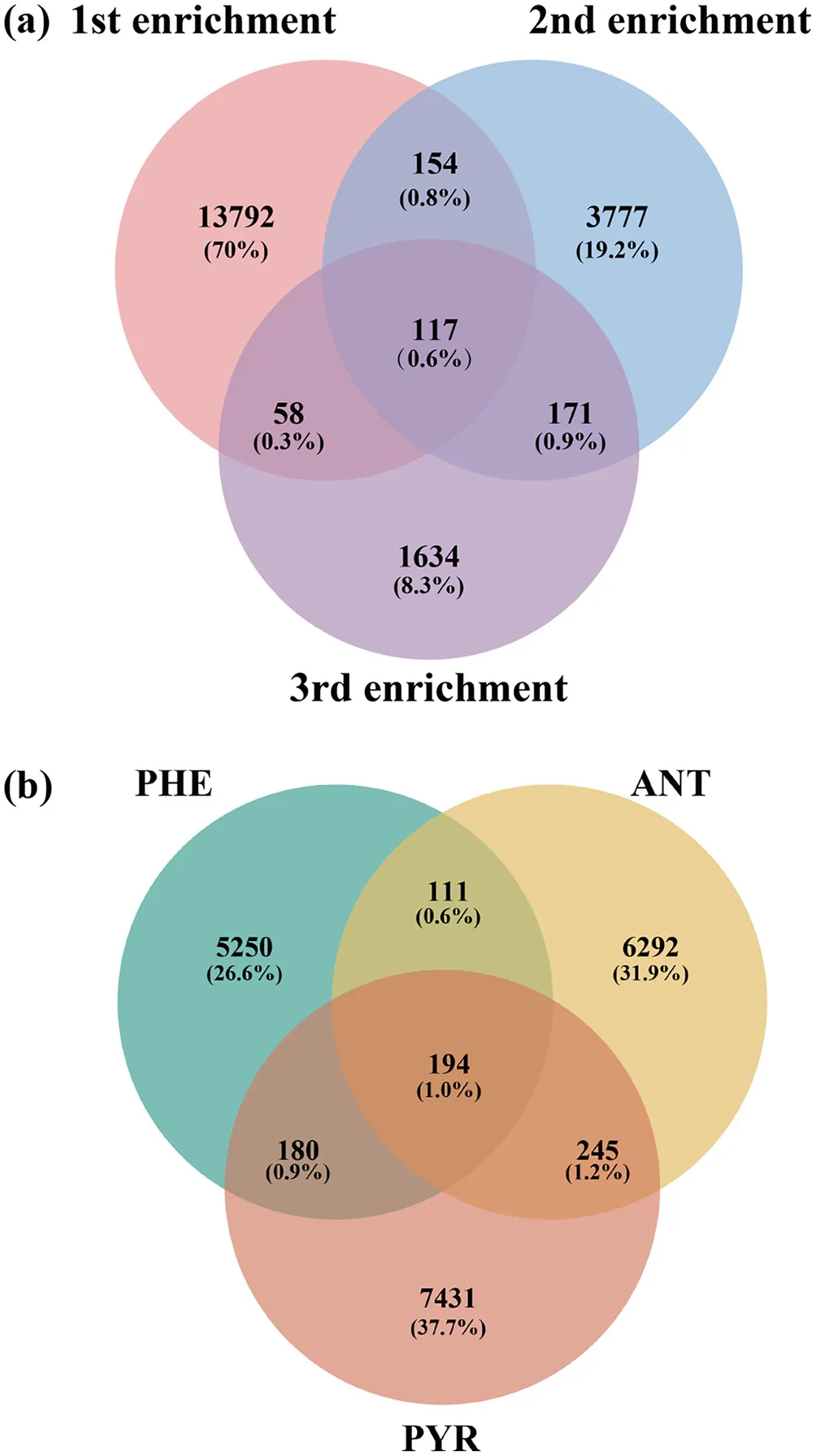
Two Venn diagrams illustrating the shared and unique ASVs among different enrichment cycles or different PAH substrates.

### 3.4 Isolation and Identification of 17 PAH‐Tolerant Strains

After the third enrichment cycle, 17 bacterial isolates able to grow on PAHs as target carbon substrates were obtained from enrichment cultures, with their detailed information listed in Table 1. Phylogenetic analysis indicated that these isolates belonged to three classes within the phylum Pseudomonadota (Figure 5). Among them, 6 strains belonged to Alphaproteobacteria, dominated by the genera *Azospirillum*, *Labrys*, *Sphingobium*, and *Sphingomonas*. Betaproteobacteria included 10 strains, mainly represented by *Burkholderia*, *Paenacidovorax*, and *Cupriavidus*. Only one strain belonged to Gammaproteobacteria and was classified into the genus *Acinetobacter*. Collectively, the isolated PAH‐tolerant strains exhibited high taxonomic diversity.

**TABLE 1:** Strain name, isolation site, utilizable PAH substrates, NCBI reference sequence, percent identity, accession ID, and taxonomic classification of the 17 PAH‐degrading bacterial strains isolated from surface sediments of the Maowei Sea mangrove wetland.

Strain name	Isolation site	Utilizable PAH substrates	NCBI reference sequence	Percent identity (%)	Accession ID	Taxonomic classification
B2⑧	MWH2	Pyrene	*Paenacidovorax caeni* strain BP6 16S ribosomal RNA gene	99.9	PX733930.1	Bacteria; Pseudomonadati; Pseudomonadota; Betaproteobacteria; Burkholderiales; Comamonadaceae; Paenacidovorax
B1⑤	MWH1	Pyrene	*Acinetobacter* sp. strain 115 16S ribosomal RNA gene	100.0	OK632279.1	Bacteria; Pseudomonadati; Pseudomonadota; Gammaproteobacteria; Moraxellales; Moraxellaceae; Acinetobacter; unclassified Acinetobacter
B5⑤	MWH5	Pyrene	*Burkholderia* sp. DB42 16S ribosomal RNA gene	99.8	JN975200.1	Bacteria; Pseudomonadati; Pseudomonadota; Betaproteobacteria; Burkholderiales; Burkholderiaceae; Burkholderia; unclassified Burkholderia
B3⑦	MWH3	Pyrene	*Cupriavidus necator* strain GX_5 16S ribosomal RNA gene	100.0	MF959436.1	Bacteria; Pseudomonadati; Pseudomonadota; Betaproteobacteria; Burkholderiales; Burkholderiaceae; Cupriavidus
N1②	MWH1	Anthracene	*Sphingobium* sp. JW16.2a partial 16S rRNA gene	99.6	FN556564.1	Bacteria; Pseudomonadati; Pseudomonadota; Alphaproteobacteria; Sphingomonadales; Sphingobiaceae; Sphingobium; unclassified Sphingobium
N2⑮	MWH2	Anthracene	*Sphingomonas* sp. M4‐VN1‐10 W gene for 16S rRNA	99.7	AB299572.1	Bacteria; Pseudomonadati; Pseudomonadota; Alphaproteobacteria; Sphingomonadales; Sphingomonadaceae; Sphingomonas; unclassified Sphingomonas
N2⑫	MWH2	Anthracene	*Sphingobium* sp. R3‐66 16S ribosomal RNA gene	99.9	JQ659638.1	Bacteria; Pseudomonadati; Pseudomonadota; Alphaproteobacteria; Sphingomonadales; Sphingobiaceae; Sphingobium; unclassified Sphingobium
N5④	MWH5	Anthracene	*Burkholderia* sp. strain SYK43 16S ribosomal RNA gene	99.7	PV424361.1	Bacteria; Pseudomonadati; Pseudomonadota; Betaproteobacteria; Burkholderiales; Burkholderiaceae; Burkholderia; unclassified Burkholderia
F3①	MWH3	Phenanthrene	*Labrys* sp. strain V61 16S ribosomal RNA gene	99.9	MT756188.1	Bacteria; Pseudomonadati; Pseudomonadota; Alphaproteobacteria; Hyphomicrobiales; Xanthobacteraceae; Labrys; unclassified Labrys
F3④	MWH3	Phenanthrene	*Azospirillum* sp. strain RS252 16S ribosomal RNA gene	99.2	PZ007824.1	Bacteria; Pseudomonadati; Pseudomonadota; Alphaproteobacteria; Rhodospirillales; Azospirillaceae; Azospirillum; unclassified Azospirillum
F7⑤	MWH4	Phenanthrene	*Burkholderia* sp. strain YH4‐13 16S ribosomal RNA gene	99.7	PZ232039.1	Bacteria; Pseudomonadati; Pseudomonadota; Betaproteobacteria; Burkholderiales; Burkholderiaceae; Burkholderia; unclassified Burkholderia
F2⑧	MWH2	Phenanthrene	*Burkholderia* sp. strain S317T 16S ribosomal RNA gene	99.7	PP542126.1	Bacteria; Pseudomonadati; Pseudomonadota; Betaproteobacteria; Burkholderiales; Burkholderiaceae; Burkholderia; unclassified Burkholderia
F2①	MWH2	Phenanthrene	*Burkholderia* sp. strain nsu‐39 16S ribosomal RNA gene	99.9	OR392921.1	Bacteria; Pseudomonadati; Pseudomonadota; Betaproteobacteria; Burkholderiales; Burkholderiaceae; Burkholderia; unclassified Burkholderia
F1⑧	MWH1	Phenanthrene	*Sphingobium* sp. NS7 gene for 16S ribosomal RNA	100.0	LC336778.1	Bacteria; Pseudomonadati; Pseudomonadota; Alphaproteobacteria; Sphingomonadales; Sphingobiaceae; Sphingobium; unclassified Sphingobium
F5⑧	MWH5	Phenanthrene	*Burkholderia* sp. strain PSCZ2 16S ribosomal RNA gene	99.9	MW048896.1	Bacteria; Pseudomonadati; Pseudomonadota; Betaproteobacteria; Burkholderiales; Burkholderiaceae; Burkholderia; unclassified Burkholderia
F5③	MWH5	Phenanthrene	*Burkholderia* sp. enrichment culture clone C4 16S ribosomal RNA gene	99.9	FJ791164.1	Bacteria; Pseudomonadati; Pseudomonadota; Betaproteobacteria; Burkholderiales; Burkholderiaceae; Burkholderia; environmental samples
F4④	MWH4	Phenanthrene	*Burkholderia* sp. Bi28MC_S_vitro 16S ribosomal RNA gene	99.9	KU242608.1	Bacteria; Pseudomonadati; Pseudomonadota; Betaproteobacteria; Burkholderiales; Burkholderiaceae; Burkholderia; unclassified Burkholderia

**FIGURE 5:**
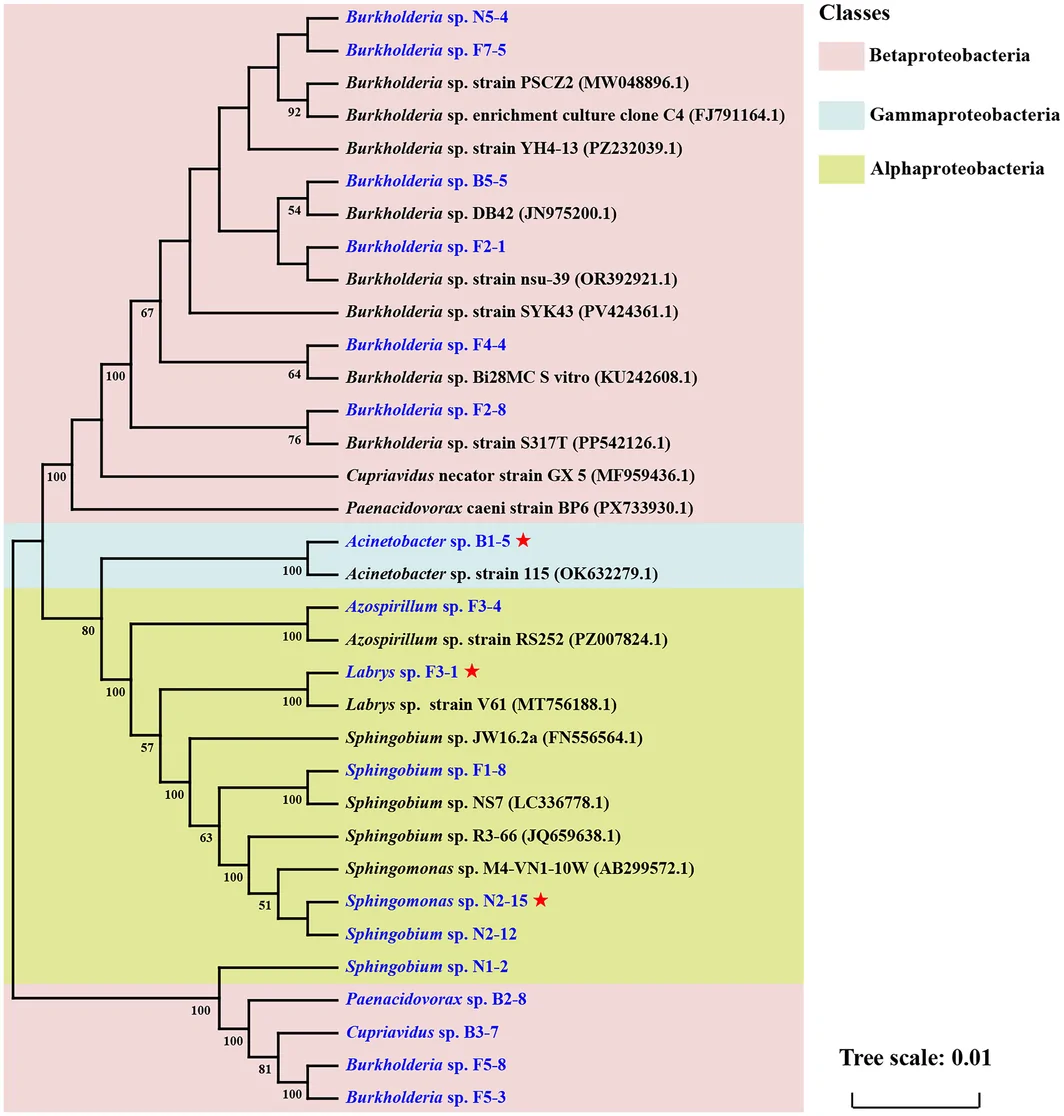
Phylogenetic tree of the 17 isolated PAH‐utilizing bacterial strains and their closely related NCBI reference sequences based on 16S rRNA gene sequences. Bootstrap values (≥ 50%) are shown at the nodes. Representative strains capable of utilizing PHE, ANT, and PYR, respectively, are marked with red asterisks. Different coloured backgrounds represent different classes of Pseudomonadota.

Among all isolated strains, *Burkholderia* (8 strains) exhibited the broadest substrate range and collectively could utilize PHE, ANT, and PYR (though individual strains varied in their substrate preferences). By contrast, *Acinetobacter* (1 strain), *Paenacidovorax* (1 strain), and *Cupriavidus* (1 strain) could only utilize PYR. *Azospirillum* (2 strains) and *Labrys* (1 strain) grew on PHE alone, whereas *Sphingobium* (2 strains) could utilize both PHE and ANT. Moreover, *Sphingomonas* (1 strain) was exclusively capable of ANT utilization. 17 pure strains shared 99.2%–100.0% similarity with known reference sequences from the NCBI database (Figure 5 and Table 1). The 16S rRNA gene sequences of the isolates were subjected to BLASTN alignment against the NCBI GenBank database. Isolates showing ≥ 97% sequence identity were identified down to the species level, while those with identities ranging from 90% to 96.9% were classified at the genus level. We adopted the NCBI database to unify the analytical workflow and access ample reference sequences of mangrove indigenous bacteria deposited in this database.

BLAST analysis revealed that *Labrys* sp. F3‐1, *Sphingomonas* sp. N2‐15, and *Acinetobacter* sp. B1‐5 exhibited 16S rRNA homology values of 99.9%, 99.7%, and 100.0% with NCBI references, respectively. These three strains, belonging to distinct genera and displaying distinct PAH utilization profiles, were selected as representative isolates to reflect the taxonomic diversity of the recovered bacteria. They were capable of metabolizing PHE, ANT, and PYR as their respective target carbon substrates, and their growth was systematically assessed under different pH (5, 7, 9, 11), temperature (4°C, 25°C, 40°C), and salinity (4‰, 8‰, 15‰, 30‰) conditions.

The results indicated that *Labrys* sp. F3–1 grew at 4°C–40°C (optimum: 40°C), pH 5.0–11.0 (optimum: pH 5.0), and salinity of 4‰–30‰ (optimum: 15‰; Figure 6a–c), and was able to utilize PHE as its target carbon source (Figure 6d).

**FIGURE 6:**
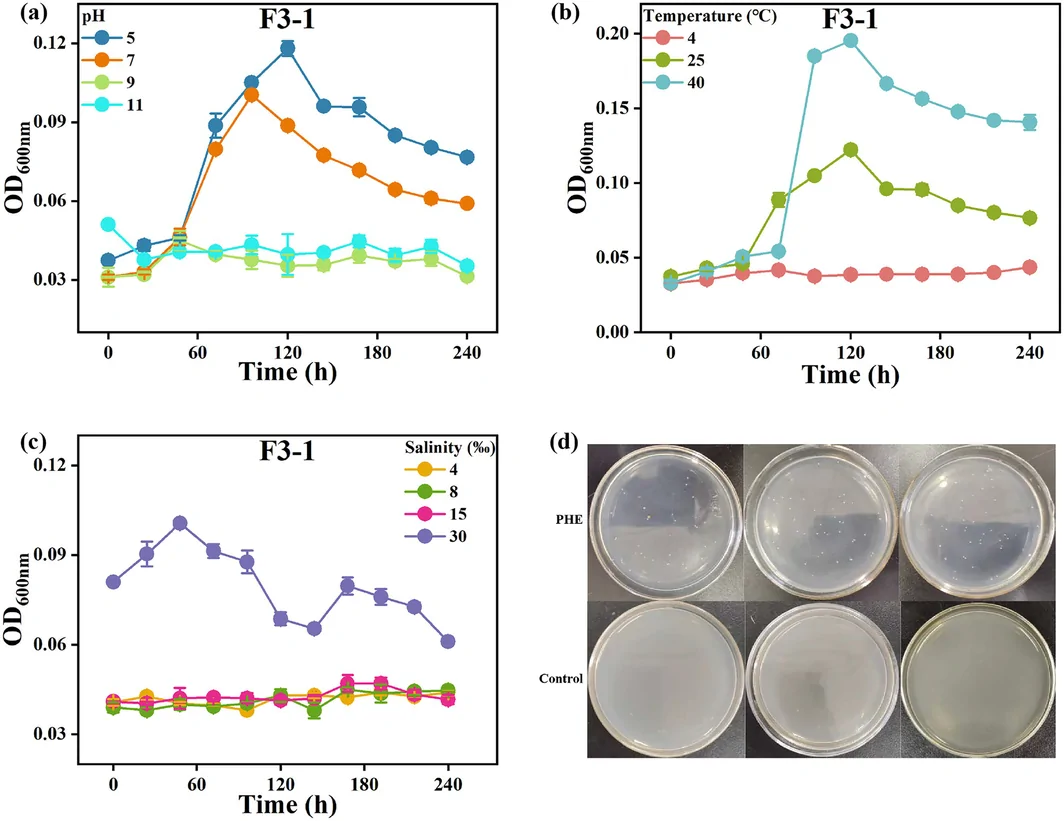
Growth curves of *Labrys* sp. F3‐1 in mineral salt medium (MSM) with PHE as the sole carbon source under different pH (a), temperatures (b), and salinity concentrations (c). (d) Growth of *Labrys* sp. F3‐1 on PHE. “PHE group” indicates MSM agar plates with PHE as the sole carbon source, while “Control group” represents MSM agar plates without PHE after 1 week of incubation. Each treatment was performed in triplicate. The sharp OD_600_ increase at 40°C reflects rapid proliferation, as this temperature is within the strains' thermal tolerance range. The elevated initial OD_600_ at 0 h under 30‰ salinity arises from the high‐salinity culture system rather than a higher starting bacterial concentration. Curves with persistently low OD_600_ (0.01–0.06) indicate strong growth inhibition, with values marginally above the blank control background.

For *Sphingomonas* sp. N2–15, it survived and proliferated at 4°C–40°C (optimum: 25°C), pH 5.0–11.0 (optimum: pH 5.0), and salinity ranging from 4‰ to 30‰ (optimum: 4‰; Figure 7a–c). Further assays confirmed that ANT could serve as its target carbon source for growth (Figure 7d).

**FIGURE 7:**
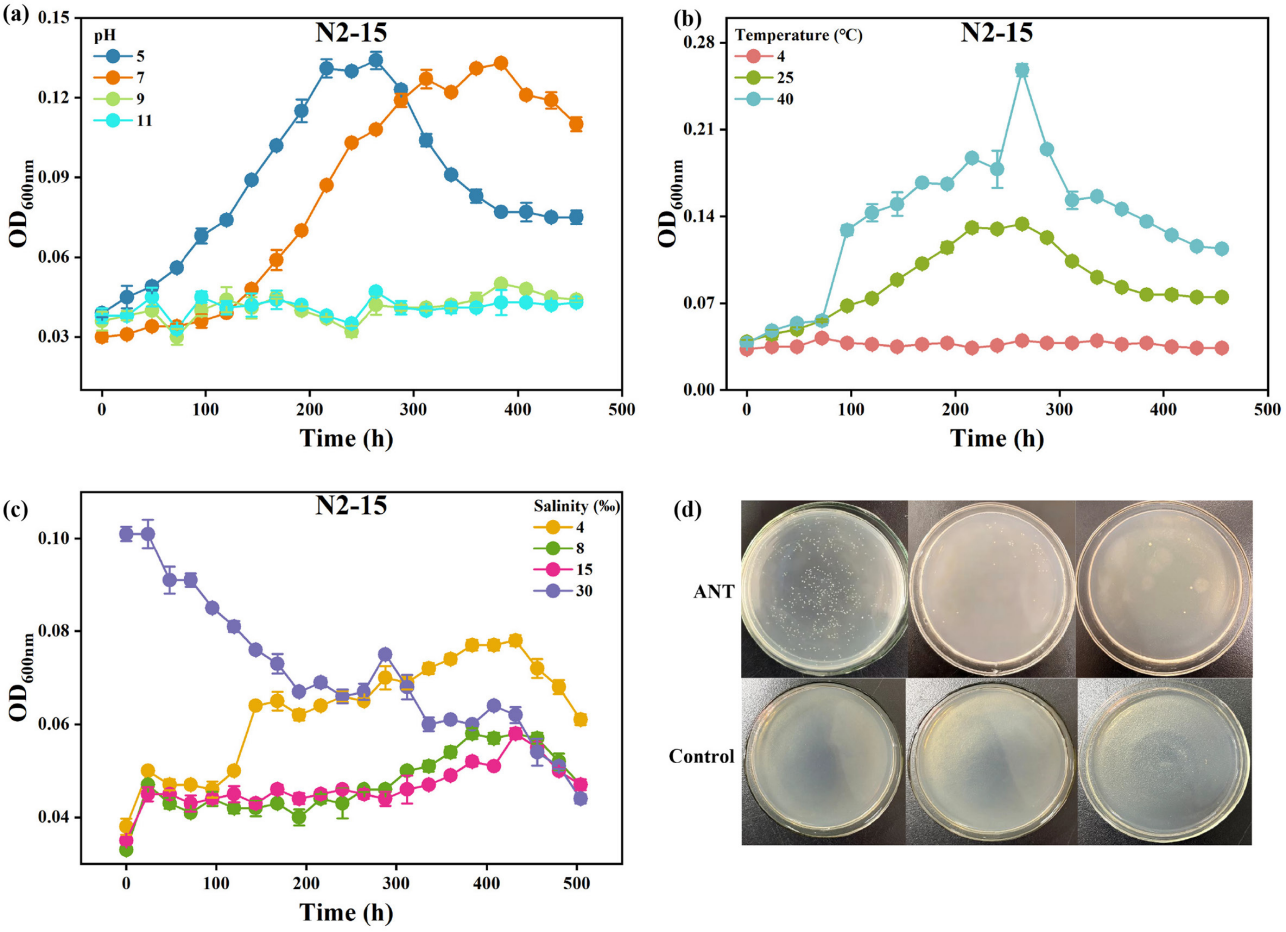
Growth curves of *Sphingomonas* sp. N2‐15 in MSM with ANT as the sole carbon source under different pH (a), temperatures (b), and salinity concentrations (c). (d) Growth of *Sphingomonas* sp. N2‐15 on ANT. “ANT group” indicates MSM agar plates with ANT as the sole carbon source, while “Control group” represents MSM agar plates without ANT after 1 week of incubation. Each treatment was performed in triplicate. The sharp OD_600_ increase at 40°C reflects rapid proliferation, as this temperature is within the strains' thermal tolerance range. The elevated initial OD_600_ at 0 h under 30‰ salinity arises from the high‐salinity culture system rather than a higher starting bacterial concentration. Curves with persistently low OD_600_ (0.01–0.06) indicate strong growth inhibition, with values marginally above the blank control background.

As for *Acinetobacter* sp. B1–5, it proliferated across 4°C–40°C (optimum: 40°C), pH 5.0–11.0 (optimum: pH 5.0), and salinity of 4‰–30‰ (optimum: 15‰; Figure 8a–c), and the isolate was capable of using PYR for growth (Figure 8d).

**FIGURE 8:**
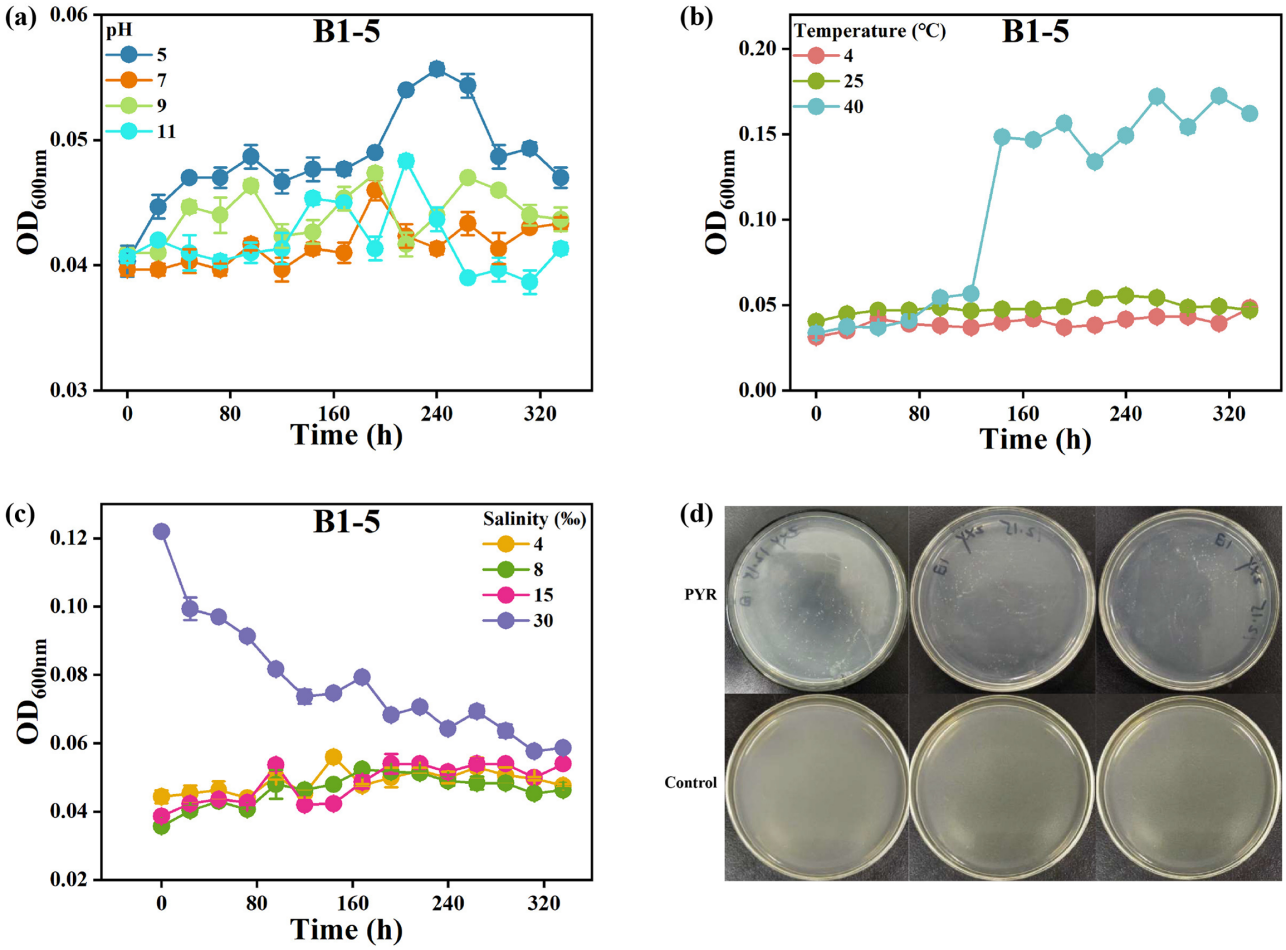
Growth curves of *Acinetobacter* sp. B1‐5 in MSM with PYR as the sole carbon source under different pH (a), temperatures (b), and salinity concentrations (c). (d) Growth of *Acinetobacter* sp. B1‐5 on PYR. “PYR group” indicates MSM agar plates with PYR as the sole carbon source, while “Control group” represents MSM agar plates without PYR after 1 week of incubation. Each treatment was performed in triplicate. The sharp OD_600_ increase at 40°C reflects rapid proliferation, as this temperature is within the strains' thermal tolerance range. The elevated initial OD_600_ at 0 h under 30‰ salinity arises from the high‐salinity culture system rather than a higher starting bacterial concentration. Curves with persistently low OD_600_ (0.01–0.06) indicate strong growth inhibition, with values marginally above the blank control background.

## 4 Discussion

### 4.1 Community Diversity and Succession of PAH‐Tolerant Microbial Consortia

Community diversity and richness declined markedly across successive transfers in the PAH‐amended enrichment cultures using PHE, ANT, and PYR as target carbon sources (Figure 2). Our findings are consistent with earlier observations on microbial community shifts during PAH‐directed enrichment cultivation (Bonaglia et al. 2020; Wang et al. 2022; Xu et al. 2022; Yu et al. 2022). For instance, Bonaglia et al. observed that the microbial alpha diversity (Shannon index) gradually decreased in oil‐contaminated sediments from Sweden during enrichment with naphthalene as the sole carbon source (Bonaglia et al. 2020). Similarly, Xu et al. revealed that microbial diversity in Karamay Oilfield soils declined with phenanthrene concentrations increasing from 100 to 300 mg/L (Xu et al. 2022). Furthermore, two independent studies revealed that microbial diversity in soils from the Yanchang Oilfield decreased after supplementation with pyrene or crude oil, compared with the initial communities (Wang et al. 2022; Yu et al. 2022).

Nevertheless, microbial diversity responds differently to PAH pollution in other investigations, depending on sample sources, substrate types, and incubation time (Qin et al. 2020; Nieto et al. 2025). As reported previously, Nieto et al. noted that microbial alpha diversity in non‐contaminated soil dropped at the early stage of a 58‐day enrichment in PAH‐supplemented microcosms and gradually converged over time (Nieto et al. 2025). Qin et al. revealed that microbial diversity and richness in gas station soils surpassed values found in initial soils after enrichment with 0.5 g/L phenanthrene as the sole carbon source (Qin et al. 2020).

The decline in microbial diversity can be mainly attributed to the following two key factors: (1) PAHs exert inherent toxicity and suppress susceptible indigenous microbes (Vagi et al. 2021; Xu et al. 2026); (2) When PAHs are used as the sole carbon source for cultivation, only a limited number of PAH‐tolerant microbes undergo selective enrichment (Shen et al. 2023; Lu et al. 2024; Sun et al. 2025). It should be noted that repeated subculturing and laboratory adaptation also contribute to diversity loss, and our experimental design cannot disentangle these confounding effects.

At the phylum level, Pseudomonadota, Bacillota and Actinomycetota dominated the PAH‐enriched mangrove sediment communities across enrichment cycles and substrate treatments (Figure 3). NMDS ordination demonstrated clear divergence of microbial communities among PAH substrates or enrichment cycles (*p* < 0.01) (Figure 2b). Likewise, numerous previous investigations have shown that the universal dominance of Pseudomonadota and Actinomycetota in PAH‐degrading microbial communities across various global habitats, such as heavily oil‐contaminated soils (Qin et al. 2020; Zhang, Manzoor, et al. 2021; Zhang, Qiu, et al. 2021; Yu et al. 2022), polluted coastal sediments (Ahmad et al. 2019; Patel et al. 2021), mangrove sediments (Meng et al. 2024), and Antarctic soils (Naloka et al. 2024).

In particular, Huang et al. (2023) demonstrated that Pseudomonadota and Actinomycetota together accounted for nearly 95% of cultivable PAH‐degrading microorganisms isolated from various habitats (Huang et al. 2023). In accordance with our results, Meng et al. found that these two phyla, Pseudomonadota (73.71%) and Actinomycetota (11.64%), dominated pyrene‐degrading communities in mangrove sediments of South China (Meng et al. 2024). Numerous previous investigations have further confirmed the predominance of Pseudomonadota in PAH‐enriched cultures. For instance, when phenanthrene or pyrene was used as the sole carbon source, Pseudomonadota became the absolute dominant phylum in microbial communities from petrochemical‐contaminated soils in Tianjin (Zhang, Manzoor, et al. 2021; Zhang, Qiu, et al. 2021). Qin et al. also reported a distinct microbial community shift in soils collected near a petrol station: the dominant phylum changed from Bacillota (99.91%) in initial soils to Pseudomonadota (93.24%–95.10%) after phenanthrene enrichment (Qin et al. 2020). Likewise, Pseudomonadota (93.4%–99.6%) prevailed in microbial consortia from oil‐polluted deep sediments in India after enrichment with naphthalene, phenanthrene, fluoranthene, and pyrene (Patel et al. 2021). In contrast, Actinomycetota became the exclusive dominant phylum in Nanjing farmland topsoil after enrichment with the exogenous PAHs naphthalene, phenanthrene and pyrene (Zhou et al. 2025).

At the family rank, the enriched consortia were mainly represented by Sphingomonadaceae, Burkholderiaceae, and Azospirillaceae (Figure 3). In accordance with our observations, Huang et al. summarized that Sphingomonadaceae, Pseudomonadaceae, and Nocardiaceae constituted the majority of PAH‐degrading microbial communities (Huang et al. 2023). Conversely, distinct dominant bacterial families have been reported in other PAH‐contaminated environments. Xu et al. demonstrated that Pseudomonadaceae, Moraxellaceae, and Micrococcaceae comprised the dominant families in phenanthrene‐degrading communities isolated from Karamay Oil Field soils (Xu et al. 2022). In parallel, Qin et al. revealed a significant shift in dominant bacterial families in soils collected near a petrol station: the predominant taxa changed from Planococcaceae (99.91%) in the original soils to Pseudomonadaceae (up to 95.10%) after phenanthrene enrichment (Qin et al. 2020).

Genus‐level profiling highlighted *Sphingobium*, *Caballeronia‐Paraburkholderia*, and *Azospirillum* as the core enriched taxa (Figure 3). Consistent with our experimental outcomes, Zhang, Manzoor, et al. (2021); Zhang, Qiu, et al. (2021) reported that *Sphingobium* (65%) and *Pseudomonas* (33%) emerged as the dominant genera in Tianjin's petrochemically contaminated soils after enrichment with phenanthrene or pyrene (Zhang, Manzoor, et al. 2021; Zhang, Qiu, et al. 2021). By contrast, Gu et al. found that *Streptomyces*, *Rhodococcus*, and *Nocardioides* formed the core PAH‐utilizing microbial consortia in landfill soils amended with phenanthrene at 50 mg/kg (Gu et al. 2023).

Overall, Pseudomonadota and Actinomycetota exhibited strong PAH‐degrading capacity. Differences in their community compositions were mainly attributed to sample origin (Kuc et al. 2019; Naloka et al. 2024; Lian et al. 2025; Peng et al. 2026), PAH substrate types (Yu et al. 2022), and incubation time (Nieto et al. 2025).

### 4.2 Isolation, Identification and Growth Adaptation of PAH‐Tolerant Bacterial Strains

Phylogenetic analysis indicated that the 17 isolates belonged to the phylum Pseudomonadota, covering three classes: Alphaproteobacteria, Betaproteobacteria, and Gammaproteobacteria. These strains were further assigned to eight genera, such as *Burkholderia*, *Acinetobacter*, and *Labrys*. Nine isolates were able to utilize PHE for sole carbon nutrition, while four strains utilized ANT and another four used PYR (Figure 5 and Table 1).

Some genera of the isolates obtained in this study have been previously recorded to grow in PAH‐amended culture systems in published studies (Al Farraj et al. 2021; Zhang et al. 2022; Ma et al. 2025). For example, Burkholderia sp. FM‐2 can grow with phenanthrene supplied as the sole carbon source. The gentisate pathway, together with key enzymes including PAH dioxygenase and extradiol dioxygenase, plays a vital role in this process (Liu et al. 2019; Ma et al. 2025). *Burkholderia vietnamiensis* G4 achieves growth on benzo[a]pyrene relying on the phenylacetic acid pathway, where the functional genes *paaN*, *paaD*, and *paaK* exert important regulatory effects (Cauduro et al. 2020). Similarly, two *Acinetobacter* strains, namely *A. baumannii* BJ5 and *A. johnsonii* , can utilize PYR and ANT, respectively, via catabolic pathways consisting of phthalic acid‐catechol and ester‐mediated branches (Jiang et al. 2018; Gupta et al. 2020; Kaur et al. 2025). Moreover, *Sphingomonas* sp. KSU05 shows adaptive growth on ANT via the phthalate‐type pathway (Al Farraj et al. 2021). Meanwhile, *Sphingobium* sp. SHPJ‐2 can sustain growth on PHE through the salicylate pathway (Zhang et al. 2022).

In addition, numerous bacterial strains other than those isolated in this study can grow with diverse PAHs as the sole carbon source under laboratory verification conditions. For example, *Pseudomonas stutzeri* PheN2 and *Pseudomonas* sp. SHPJ 1 show high efficiencies in phenanthrene degradation (Zhang et al. 2022, 2023). Strains belonging to *Pseudomonas* and *Stutzerimonas* can also efficiently remove phenanthrene and benzo[a]pyrene simultaneously in nitrate‐reducing environments (Zhao et al. 2026). *Streptomyces* sp. M‐1 and *Bacillus licheniformis* are capable of utilizing ANT and PHE, respectively, whereas *Mycolicibacterium* sp. PAM1 has been shown to use both PHE and PYR (Golubev et al. 2021; Hoque et al. 2023; Zhang, Li, et al. 2025; Zhang, Qian, et al. 2025). Further research revealed that *Mycolicibacterium* sp. PAM1 depends on the genes *nidA* and *nidB* to metabolize PHE (Golubev et al. 2021). By contrast, *Pelagerythrobacter* sp. N7 mineralizes PHE via a combination of the catechol, gentisate and phthalate pathways (Li, Cao, et al. 2024; Li, Huang, et al. 2024). *Stenotrophomonas maltophilia* CPHE1 degraded PHE through convergent phthalate and salicylate pathways (Lara‐Moreno et al. 2021). Moreover, *Achromobacter xylosoxidans* BUK_BTEG6 utilizes ANT via the canonical dioxygenase‐initiated *ortho*‐cleavage pathway (Usman et al. 2023).

Notably, the three representative strains exhibited divergent physiological optima under gradients of pH, temperature, and salinity, yet all could maintain growth under PAH selective pressure, highlighting their adaptive potential across fluctuating environmental conditions (Figures 6, 7, 8).

To tentatively infer potential PAH transformation‐related metabolic pathways of strains F3‐1, N2‐15 and B1‐5 based on published evidence, we summarized relevant previous researches. Few investigations have documented verified PAH biotransformation capacity within the genus *Labrys*. Nevertheless, Csépányi et al. reported that *Labrys sedimenti* is capable of efficient diclofenac transformation relying on coupled benzoate degradation and catechol ortho‐cleavage pathways (Csépányi et al. 2025). It is therefore hypothesized that *Labrys* sp. F3‐1 may harbour analogous enzyme cascades that potentially participate in PHE transformation. Secondly, GC–MS quantification in an earlier study verified that *Sphingomonas* sp. KSU05 can metabolize anthracene (Al Farraj et al. 2021), whereas the systematic PAH transformation pathways for most members of the genus *Sphingomonas* have not been fully elucidated. Furthermore, validated studies demonstrated that *Acinetobacter baumannii* BJ5 transforms PYR and PHE via two independent metabolic routes: one is the phthalic acid pathway producing catechol, and the other proceeds via benzyl benzoate intermediates to generate short‐chain esters (Gupta et al. 2020, Kaur et al. 2025). Another strain *Acinetobacter* sp. YY‐1 can facilitate the transformation of PYR and PHE mediated by quorum sensing (QS) systems controlled by the *rhlI* and *sdiA* genes (Huang et al. 2025). On this basis, we speculate that *Acinetobacter* sp. B1‐5 may possess similar metabolic machinery to mediate possible pyrene transformation under suitable culture conditions.

Different microorganisms degrade various PAHs, which is mainly determined by their molecular structures and bioavailability. For instance, high‐molecular‐weight (HMW) PAHs such as pyrene rely on more complex and synergistic enzymatic systems to achieve degradation (Davletgildeeva and Kuznetsov 2024). Furthermore, most PAH‐degrading bacteria belong to the phylum *Pseudomonadota*, reflecting their strong tolerance and adaptability to PAH contamination (Hua et al. 2023). Taken together, our results reveal that the PAH‐tolerant bacterial strains obtained in this study, particularly the metabolically versatile genus *Burkholderia*, can serve as potential candidate resources for subsequent research on bioremediation of PAH‐contaminated mangrove sediments (Morya et al. 2020; Ortega Ramirez et al. 2020; Liu et al. 2025).

This study has several limitations. Although isolates grew stably with PAHs as the sole carbon source (colony and OD data), GC–MS quantification of residual PAHs and metabolite identification are ongoing, and PAH‐dependent growth only proves tolerance rather than confirmed biodegradation or mineralization. We have softened overstated degradation claims, and predicted PAH metabolic pathways need experimental validation. The tested PAH concentrations (100–500 mg/L) are laboratory‐optimized and higher than mangrove sediment background levels, so the observed high tolerance may not hold at environmentally relevant low concentrations; further low‐dose incubation, PAH quantification and metabolite analysis are required to evaluate biotransformation and field potential. The high recovery of *Burkholderia* should be interpreted cautiously, as 16S rRNA sequencing only offers genus‐level identification and cannot resolve intraspecific variation; its dominance likely stems from artificial PAH selection, and future WGS, MLST, or rep‐PCR will clarify intraspecific diversity and functional redundancy. Physiological gradients of pH, temperature, and salinity only compare tolerance under constant PAH stress instead of defining full thresholds, and the weakly acidic optimum in pure culture is stress‐related, so we avoid overextrapolating lab results for field remediation, with on‐site validation planned. Stepwise PAH enrichment (100, 300, 500 mg/L) with repeated subculturing cannot separate effects of increasing PAH and serial transfer, and alpha‐diversity loss and community shifts result from combined selection pressures. Pearson correlation was used without post hoc pairwise tests, and extra statistical comparisons will be added later. Taxonomic annotation using NCBI GenBank suits our amplicon workflow for cross‐study comparison, while EzBioCloud cross‐checks could improve resolution. No baseline sequencing of raw sediments before enrichment limits direct comparison between enriched and indigenous communities.

## 5 Conclusion

This current investigation elucidates the successional trajectories of microbial communities under polycyclic aromatic hydrocarbon (PAH) stress in mangrove sediments of the Maowei Sea, Guangxi, China. Following three successive rounds of gradient enrichment, we found that microbial community diversity declined with prolonged enrichment and varied across different PAH substrates, accompanied by significant community divergence among treatments (*p* < 0.01). Pseudomonadota (33.2%–99.9%), Bacillota (0%–48.3%), and Actinomycetota (0.1%–33.7%) were the dominant bacterial phyla in the PAH enrichment cultures. A total of 17 indigenous PAH‐tolerant bacterial strains were isolated and classified into eight genera affiliated with Pseudomonadota, with representative isolates belonging to *Labrys*, *Sphingomonas*, and *Acinetobacter*. Under the tested physiological gradients, *Labrys* sp. F3‐1 and *Acinetobacter* sp. B1‐5 exhibited optimal growth at pH 5.0, 40°C, and 15‰ salinity, while *Sphingomonas* sp. N2‐15 showed maximum growth at pH 5.0, 25°C, and 4‰ salinity within the same tested range. Subsequent growth assays under PAH stress confirmed that these three strains could survive and proliferate in selective media supplemented with phenanthrene, anthracene, or pyrene as target carbon substrates. Taken together, the present investigation provides valuable indigenous bacterial resources and basic theoretical references for follow‐up research exploring the bioremediation potential of PAH‐contaminated mangrove sediments. Given the high enrichment concentrations used in this study, the reported community shifts and strain physiological traits should be interpreted as adaptive responses under extreme laboratory selective pressure; extrapolation to in situ mangrove microbial communities under ambient environmental PAH levels should be made with caution.

## Author Contributions

**Yunru Li:** methodology, software, data curation, investigation, writing – original draft, visualization, formal analysis. **Songze Chen:** data curation, writing – review and editing, software, methodology, formal analysis, resources. **Houxian Lan:** writing – review and editing, funding acquisition, conceptualization. **Yun Tao:** methodology, investigation. **Qing Li:** methodology, investigation. **Xian Xu:** methodology, investigation. **Ju Huang:** investigation, validation. **Ting Su:** investigation, validation. **Xiyu Zhang:** investigation, validation. **Tianqi Li:** investigation, validation. **Dan Sun:** writing – review and editing. **Mingzhong Liang:** writing – review and editing, funding acquisition. **Jingjing Song:** writing – review and editing, project administration. **Bin Gong:** writing – original draft, project administration, resources, funding acquisition, supervision, conceptualization. **Ying Liu:** conceptualization, methodology, funding acquisition, project administration, resources, writing – review and editing. **Rongping Bu:** conceptualization, supervision, funding acquisition, project administration, resources, writing – review and editing.

## Funding

This work was supported by the Natural Science Foundation of Guangxi Zhuang Autonomous Region, Project Nos. 2025GXNSFHA069226, 2024GXNSFBA010371, 2026GXNSFBA00640242, 2025GXNSFHA069232; the Beibu Gulf University (High‐level Talent Scientific Research Start‐up Project, Project No. 23KYQD18); Education Department of Guangxi Zhuang Autonomous Region (Improving the Basic Scientific Research Capability of Young and Middle‐aged Teachers in Guangxi Colleges Project, Project No. 2025KY0471); Beibu Gulf University (Marine Science and Technology Innovation Cooperation Foundation of Beibu Gulf Project, Project No. 03190010); National College Students Innovation and Entrepreneurship Training Program, Project No. S202511607100; Beibu Gulf University (Development of Utilization Technology of Probiotics in Fish Gut of Symbiotic System Project, Project No. 02040772).

## Conflicts of Interest

The authors declare no conflicts of interest.

## Supporting information


**Table S1:** Physicochemical parameters of surface sediment samples from the Maowei Sea mangrove wetland.

**Figure S1:** Variations in Shannon, ACE and Chao indices of microbial communities during three‐cycle PAH enrichment for PHE, ANT and PYR treatments. Each dot corresponds to an individual sample. Pearson correlation coefficients (R) and *p*‐values are displayed in each panel.
**Figure S2:** Acetone solvent control for the growth assay on PAHs. F3‐1, N2‐15 and B1‐5 are the acetone solvent controls corresponding to PHE, ANT, and PYR groups, respectively. “Acetone solvent control group” indicates MSM agar plates supplemented with equal volume of acetone without polycyclic aromatic hydrocarbons after 1 week of incubation. Each treatment was performed in triplicate.
**Figure S3:** Taxonomic composition of microbial communities at two levels (family and genus) after three cycles of enrichment with PHE, ANT and PYR, respectively. The top 10 taxa with the highest relative abundance are shown at the family and genus levels. Sample designation format: [carbon source code]‐[sampling site]‐[enrichment cycle]. F = PHE, N = ANT, B = PYR; 1–5 = sampling sites 1–5; −1 to −3 = first to third 1‐week enrichment cycles.

## Data Availability

All raw sequence datasets of 16S rRNA genes from this study have been deposited into the NCBI Sequence Read Archive database with accession No. PRJNA1465603.
