# Supplementary material for: Succession of Microbial Communities and Identification of Indigenous PAH‐Tolerant Bacteria Under Multiple PAH Stresses in Mangrove Sediments From Maowei Sea, Guangxi, China
Source: Microb Biotechnol. 2026 Nov 10;19(11):e70467. doi: 10.1111/1751-7915.70467 (PMC13752475; doi:10.1111/1751-7915.70467)
Supplement: Supplementary file 1 [file MBT2-19-e70467-s001.zip › mbt270467-sup-0002-Figure S1.docx]

**Figure legend**

**FIGURE S1** Variations in Shannon, ACE and Chao indices of microbial communities during three-cycle PAH enrichment for PHE, ANT and PYR treatments. Each dot corresponds to an individual sample. Pearson correlation coefficients (R) and P-values are displayed in each panel.

**FIGURE S2** Acetone solvent control for the growth assay on PAHs. F3-1, N2-15 and B1-5 are the acetone solvent controls corresponding to PHE, ANT, and PYR groups, respectively. “Acetone solvent control group” indicates MSM agar plates supplemented with equal volume of acetone without polycyclic aromatic hydrocarbons after one week of incubation. Each treatment was performed in triplicate.

**FIGURE S3** Taxonomic composition of microbial communities at two levels (family and genus) after three cycles of enrichment with PHE, ANT and PYR, respectively. The top 10 taxa with the highest relative abundance are shown at the family and genus levels. Note: sample designation format: [carbon source code]- [sampling site]- [enrichment cycle]. F = PHE, N = ANT, B = PYR; 1–5 = sampling sites 1–5; -1 to -3 = first to third one-week enrichment cycles.
